# Genetic yield of next-generation sequencing for detecting monogenic familial hypercholesterolemia in uzbek patients with coronary artery disease

**DOI:** 10.1371/journal.pone.0353401

**Published:** 2026-07-09

**Authors:** Rano B. Alieva, Aleksandr B. Shek, Anastasiya V. Bahachova, Khurshid G. Fozilov, Guzal J. Abdullaeva, Alisher A. Abdullaev, Lilya E. Kan, Shavkat U. Khoshimov, Andrey R. Kim, Ulugbek I. Nizamov, Dilnora B. Yusupalieva

**Affiliations:** 1 Republican Specialized Scientific and Practical Medical Center for Cardiology, Ministry of Health of the Republic Uzbekistan, Tashkent, Uzbekistan; 2 Independent Consultant, Tbilisi, Georgia; 3 Center for Advanced Technologies, Ministry of Higher Education, Science and Innovation of the Republic Uzbekistan, Tashkent, Uzbekistan; Odense University Hospital, DENMARK

## Abstract

**Background:**

Familial hypercholesterolaemia (FH) is an inherited disorder with markedly elevated LDL-C and increased risk of premature atherosclerotic cardiovascular disease, most often caused by pathogenic variants in *LDLR* and less frequently *APOB*/*PCSK9* (or recessive *LDLRAP1*). FH is commonly assessed using the Dutch Lipid Clinic Network (DLCN) score (definite >8, probable 6–8, possible 3–5). In Uzbekistan, genetic evidence for FH remains limited and largely based on candidate-variant studies, and the diagnostic yield of NGS for monogenic FH in CAD patients is not well defined.

**Aim:**

For the first time in Uzbekistan and Central Asia, to investigate FH-associated monogenic variants using next-generation sequencing (NGS) and to assess the validity of the DLCN criteria against genetic testing as the diagnostic reference standard in Uzbek patients with CAD and suspected FH.

**Methods:**

This study included 95 patients with coronary artery disease (CAD) who underwent targeted NGS of *LDLR*, *APOB*, *PCSK9*, and *LDLRAP1*. The suspected/phenotypic FH group comprised 56 patients: 53 with DLCN-predicted heterozygous FH (HeFH)—possible (3–5 points, n = 22), probable (6–8 points, n = 16), and definite (>8 points, n = 15)—and 3 siblings from one family with a homozygous FH (HoFH) phenotype. The control group included 39 CAD patients with hypercholesterolemia without an FH diagnosis (DLCN 1–2 points). Only pathogenic/likely pathogenic (P/LP) variants were used for genetic confirmation of FH.

**Results:**

Pathogenic/likely pathogenic variants were detected in 10/53 (18.9%) DLCN-predicted HeFH patients and in all three HoFH siblings. Genetic confirmation rates (PPV) were 46.7% (7/15) in definite HeFH, 12.5% (2/16) in probable HeFH, and 4.5% (1/22) in possible HeFH; no P/LP variants were detected in controls (0/39). Using a DLCN >8 threshold, sensitivity was 70.0% (7/10) and specificity was 90.2% (74/82) in the CAD cohort excluding the HoFH family.

**Conclusion:**

NGS confirmed the highest diagnostic yield in patients with DLCN >8, supporting its use as a practical threshold to prioritise genetic testing; however, monogenic FH may still be present in patients with probable or possible DLCN scores.

## 1. Introduction

Familial hypercholesterolemia (FH) is a prevalent autosomal-dominant genetic cause of premature coronary artery disease, driven by lifelong elevated low-density lipoprotein cholesterol (LDL-C) levels [1,2]. If left untreated, heterozygous FH (HeFH) typically leads to premature coronary artery disease before the age of 55 years, whereas in homozygous FH (HoFH) coronary artery disease usually develops very early and, without treatment, patients may die before the age of 20 years [2–5]. According to international registries [6], FH accounts for approximately 20% of myocardial infarctions occurring each year in young working-age individuals aged 25–50 years and this aggravates the course of the disease [7].

Cardiovascular mortality in Uzbekistan reaches 61%, and more than 50% of cardiovascular deaths are attributable to atherosclerotic cardiovascular disease (ASCVD). The national healthcare system has set an ambitious goal to reduce premature cardiovascular mortality by 30% by 2030, which is closely linked to addressing dyslipidemia. Based on the average prevalence of HeFH in Europe (1:200) [8], among Uzbekistan’s population of 38 million, approximately 190,000 individuals are affected. To improve FH diagnosis and management, the Atherosclerosis Society of the Republic of Uzbekistan participates in studies within the global registries Familial Hypercholesterolaemia Studies Collaboration (FHSC) [9–12] and the Homozygous FH International Clinical Collaborators (HICC) registry [13].

However, comprehensive assessment of the full spectrum of FH genetic variants in the Uzbek population using next-generation sequencing (NGS) became possible only within the framework of the scientific grant “Development of protocols for the diagnosis and treatment of inherited dyslipidemia” (State Registration No. AL-8223092044), which provides for three sequencing rounds over a three-year period; the first-year results formed the basis of this article.

In this context, our aim was, for the first time in Uzbekistan and Central Asia, to investigate FH-associated monogenic variants using next-generation sequencing (NGS) and to assess the validity of the DLCN criteria against genetic testing as the diagnostic reference standard in Uzbek patients with CAD and suspected FH.

## 2. Materials and methods

### 2.1. Study population

A total of 95 patients with coronary artery disease (CAD) were enrolled from August 1, 2024 to April 10, 2025: 53 with heterozygous familial hypercholesterolemia (HeFH) classified by the Dutch Lipid Clinic Network (DLCN) score as possible (3–5 points, n = 22), probable (6–8 points, n = 16), or definite (>8 points, n = 15), and 3 patients (one brother and two sisters) with a clinical phenotype of homozygous familial hypercholesterolemia (HoFH), in whom CAD onset occurred at the ages of 18 and 20 years. The control group comprised 39 patients with CAD and hypercholesterolemia without an FH diagnosis (DLCN 1–2 points). In this group, LDL cholesterol levels did not exceed 190 mg/dL (1 point in the DLCN score) but could be below 155 mg/dL provided that 1–2 points were assigned for a positive family history.

Inclusion criteria were: (i) age ≥ 18 years; (ii) documented coronary artery disease (CAD); (iii) hypercholesterolemia with available LDL-C measurements (preferably untreated; otherwise highest pre-treatment or estimated untreated LDL-C for DLCN scoring); (iv) sufficient clinical information to calculate the Dutch Lipid Clinic Network (DLCN) score; and (v) written informed consent for participation and genetic testing. Exclusion criteria were: refusal to provide informed consent; insufficient data to calculate the DLCN score; and inability to obtain an adequate blood sample or DNA quality for NGS library preparation and evidence of secondary hypercholesterolemia (including untreated hypothyroidism; nephrotic syndrome or advanced renal disease; cholestatic/obstructive liver disease; uncontrolled diabetes mellitus; and use of medications known to increase LDL-C, such as systemic glucocorticoids, ciclosporin, retinoids, protease inhibitors, or thiazide diuretics).

For the purposes of this CAD-based diagnostic accuracy study, the control group comprised CAD patients with hypercholesterolemia and low clinical probability of FH (DLCN 1–2 points, i.e., “unlikely FH”), rather than healthy normolipidemic individuals, to ensure a clinically comparable reference group and reduce spectrum bias.

Demographic and clinical data (including age, sex, cardiovascular history, and routine laboratory measures shown in Table 1) were collected from medical records and structured patient interview. The DLCN score was calculated according to standard criteria using available information on family history, clinical history and examination findings, and lipid levels; genetic results were not used to assign DLCN categories.

**Table 1:** Baseline Clinical and Laboratory Characteristics.

Parameters	Controls (no FH) (n = 39)	HeFH (n = 53)	HoFH* (n = 3)	P (Controls vs HeFH)
Age	61.9 ± 11.9	56.2 ± 13.1	22.0 ± 3.6	<0.001
Men/women (%)	19/20 (48.7%/51.3%)	29/24 (54.7%/45.3%)	1/2 (33.3%/66.7%)	NS
Hypertension, n (%)	19 (48.7%)	22 (41.5%)	0 (0%)	NS
T2DM, n (%)	9 (23%)	7 (13.2%)	0 (0%)	NS
History of MI, n (%)	5 (12.8%)	10 (19%)	0 (0%)	NS
History of PCI, n (%)	6 (15.4%)	15 (28.3%)	0 (0%)	NS
History of CABG, n (%)	1 (2.6%)	6 (11.3%)	1 (33.3%)	NS
History of IS, n (%)	0 (0%)	1 (1.9%)	0 (0%)	–
History of all MACE, n (%)	12 (30.8%)	32 (60.4%)	1 (33.3%)	<0.05
BMI, kg/m2	30.0 ± 5.7	30.4 ± 6.04	21.9 ± 2.1	NS
TC, mg/dL ^	236 (210–266)	277 (253-314)	730 (673-818)	<0.001
TG, mg/dL ^	229 (118.0-255.0)	165 (120.0-221.0)	101(98.0-105.5)	NS
HDL-C, mg/dL ^	48 (38.25-56.75)	52 (40.75-62.0)	26 (18.5-31.0)	NS
LDL-C, mg/dL ^	146 (132-171)	205 (174-218)	705 (622-770)	<0.001
ApoA-I, mg/dL ^	144 (129-157)	152 (135-173)	–	NS
ApoB, mg/dL^	93 (78-109)	140 (120-157)	–	<0.001
ApoB/ApoA-I ^	37 (6.0-57.75)	41 (8.0-60.0)	87 (72.7-100.3)	NS
Lp(a), mg/dL ^	5.5 (4.7-6.8)	5.3 (5.0-6.0)	4.5 (3.9-5.0)	NS
Glucose, mmol/L ^	4 (1.4-4.7)	5 (1.0-2.9)	–	NS
PCSK9, ng/ml ^	230 (93-201)	262 (218-322)	–	NS
Insulin, μU/mL ^	19 (11.6-25.16)	13 (5.9-17.5)	–	NS
Tendon xanthomas, n (%)	0 (0.0%)	5 (9.4%)	3 (100%)	0.070†
Corneal arcus in a person <45, n (%)	0 (0.0%)	9 (17.0%)	3 (100%)	0.009†
Family history of premature CAD, n (%)	21 (53.8%)	49 (92.5%)	3 (100%)	<0.001†

Data are presented as median (25th–75th percentiles) ^ or n (%). p values are for comparisons between controls (no FH) and the HeFH subgroup. The Mann–Whitney U test was used for continuous variables, and the χ² test or Fisher’s exact test (when expected cell counts were <5) was used for categorical variables. †Fisher’s exact test was used for these categorical comparisons due to small cell counts/zero cells.

* Group 3 (HoFH, n = 3) is presented descriptively; formal statistical comparisons with the other groups were not performed due to the very small sample size and limited confidence in the estimates.

**Abbreviations**: T2DM, type 2 diabetes mellitus; MI, myocardial infarction; IS, ischemic stroke; CABG, coronary artery bypass grafting; PCI, percutaneous coronary intervention; TC, total cholesterol; TG, triglycerides; HDL-C, high-density lipoprotein cholesterol; LDL-C, low-density lipoprotein cholesterol; Apo, apolipoprotein; hsCRP, high-sensitive C-reactive protein; Lp(a), lipoprotein(a).

LDL-C values used for DLCN scoring were ideally obtained off lipid-lowering therapy (untreated). When only on-treatment LDL-C was available, we used the highest documented pre-treatment LDL-C; if unavailable, untreated LDL-C was estimated from the treated value using published drug- and dose-specific correction factors. LDL-C was then assigned DLCN points using standard cut-offs (4.0–4.9 mmol/L [155–190 mg/dL], 5.0–6.4 mmol/L [191–250 mg/dL], 6.5–8.4 mmol/L [251–325 mg/dL], and ≥8.5 mmol/L [≥326 mg/dL]).

This sampling strategy at the initial stage allowed us to: (i) evaluate the validity of the DLCN criteria by comparing possible, probable, and definite HeFH classifications with the diagnostic “gold standard” (genetic testing); (ii) assess differences between HeFH and polygenic hypercholesterolemia in Uzbek CAD patients; and (iii) verify concordance between the clinical HoFH phenotype and the genetic diagnosis.

The project topic, “Development of protocols for the diagnosis and treatment of hereditary dyslipidemia,” was approved by the Scientific Council of the Republican Specialized Scientific and Practical Medical Center of Cardiology (RSSPMC of Cardiology) on July 31, 2023 (Protocol No. 7). Ethical approval for the study was granted by the Ethics Committee of the RSSPMC of Cardiology on December 7, 2023 (Protocol No. 5); the informed consent form was approved, and all participants provided written informed consent in accordance with the Declaration of Helsinki.

### 2.2. Instrumental method

CAD was confirmed by the treating cardiologist based on medical records and objective evidence from standard clinical evaluation and coronary imaging (CT angiography and/or invasive coronary angiography) as clinically indicated. The full list of instrumental procedures used for CAD confirmation and eligibility assessment is provided in Supporting information file (S1 Text in S1 File).

### 2.3. Biochemical studies

Assessment of blood lipid profile, apolipoproteins A (apoA) and B (apoB), glucose, high-sensitivity C-reactive protein (hsCRP), and interleukin-6 was conducted using a cobas c 311 automatic biochemical analyzer (Roche Diagnostics GmbH, Mannheim, Germany) with standardized Roche test systems (Roche Diagnostics GmbH, Mannheim, Germany).

Lipoprotein(a) [Lp(a)] levels (mg/dL) in serum were determined using an immunoturbidimetric method with latex enhancement on the cobas c 311 analyzer (Roche Diagnostics GmbH, Mannheim, Germany) with latex particles coated with human Lp(a) antibodies.

Concentrations of insulin were measured using a cobas e 411 immunochemiluminescent analyzer (Roche Diagnostics GmbH, Mannheim, Germany) and standardized Roche test systems (Roche Diagnostics GmbH, Mannheim, Germany).

Proprotein convertase subtilisin/kexin type 9 (PCSK9) levels were measured by enzyme-linked immunosorbent assay (ELISA) using the Human Proprotein Convertase 9/PCSK9 ELISA Kit (Multi Sciences Biotech Co., Ltd., Hangzhou, China) according to the standard protocol.

### 2.4. Genetic analyses

#### 2.4.1. DNA extraction.

Total genomic DNA was extracted from 100 venous blood samples using the DNeasy Blood & Tissue Kit (QIAGEN, Hilden, Germany) according to the manufacturer’s instructions. DNA concentration and quality were assessed using a Quantus fluorometer (Promega Corporation, Madison, WI, USA) with the QuantiFluor ONE dsDNA System (Promega Corporation, Madison, WI, USA), and a NanoPhotometer NP80 spectrophotometer (Implen, Munich, Germany), following the manufacturers’ recommendations. For downstream analysis, 96 genomic DNA samples with an input amount/concentration of 10–80 ng, meeting the requirements of the targeted sequencing library preparation protocol, were accepted.

No adverse events related to study procedures (venous blood sampling and NGS) were recorded.

#### 2.4.2. Library preparation and targeted DNA sequencing.

To detect genetic variants in *LDLR*, *APOB*, *PCSK9*, and *LDLRAP1*, next-generation sequencing (NGS) was performed on the MiSeq platform (Illumina, San Diego, CA, USA). Library preparation was carried out using a targeted enrichment approach with a custom-designed panel, QIAseq Targeted DNA Pro Custom (96) (QIAGEN, Hilden, Germany), in accordance with the manufacturer’s protocol.

Additional manufacturer documentation and software resources are provided in Supporting information file (S2 Text in S1 File).

A detailed step-by-step laboratory workflow and sequencing protocol has also been deposited in protocols.io and is available at https://doi.org/10.17504/protocols.io.bp2l6jqbkvqe/v1.

Library preparation followed the QIAseq Targeted DNA Pro Custom protocol and included enzymatic fragmentation/end repair/A-tailing, UMI adapter ligation, and target enrichment PCR; further indexing and sequencing steps are described below and in OR1.

Panel design was completed by QIAGEN specialists upon individual request. The panel covered coding exons and flanking intronic regions (including splice sites) in *LDLR*, *LDLRAP1*, *PCSK9*, and *APOB*. Other lipid metabolism-related genes were not included because the primary aim of this study was to investigate established monogenic causes of FH. Primers were supplied as a single pooled mix for target enrichment. A BED file was also provided containing panel information, including chromosome number; amplicon start and end coordinates (GRCh38); strand orientation (+/−); and primer positions. Detailed amplicon design and primer coordinates are provided in S7 Table in S1 File.

Indexing using unique dual indexes (UDIs) with QIAseq Targeted DNA Pro UDI Set B (96) (QIAGEN, Hilden, Germany), followed by universal PCR on a C1000 Touch Thermal Cycler (Bio-Rad Laboratories, Hercules, CA, USA).

Sequencing. Libraries were diluted to 4 nM, pooled, and further diluted to a final loading concentration of 4–7 pM. Sequencing was performed on MiSeq (Illumina, San Diego, CA, USA) using MiSeq Reagent Kit v2 (300 cycles) (Illumina, San Diego, CA, USA) and PhiX Control v3 (Illumina, San Diego, CA, USA). The run configuration followed the manufacturer’s recommendations: Read 1: 149 bp; Read 2: 149 bp; Index reads: 10 bp each. Run setup and monitoring were performed using Local Run Manager (LRM) v2 (Illumina, San Diego, CA, USA).

#### 2.4.3. Data quality control and bioinformatic analysis.

Following sequencing, FASTQ files were generated for each of the 96 samples. Primary filtering and quality control were performed using built-in tools in CLC Genomics Workbench (QIAGEN, Aarhus, Denmark), including assessment of total read count, mean Q-score, and GC-content distribution.

Variant analysis was conducted using the Biomedical Genomics Analysis plugin within CLC Genomics Workbench (QIAGEN, Aarhus, Denmark). A template pipeline optimized for QIAseq targeted panels was applied, including read filtering, UMI processing, alignment, variant calling, variant filtering, and annotation. The analysis output consisted of VCF files suitable for subsequent clinical review. Variants with a minimum sequencing depth of 30× and variant allele fraction (VAF) ≥20% were considered for downstream clinical interpretation, while lower-frequency variants were reviewed manually when supported by read quality and UMI evidence.

For variant interpretation, the QIAGEN Clinical Insight (QCI) Interpret (QIAGEN, Aarhus, Denmark) platform was used to review, classify, and retrieve clinically relevant evidence for detected variants according to the American College of Medical Genetics and Genomics and the Association for Molecular Pathology (ACMG/AMP) guidelines [14]. For *LDLR*, gene-specific refinements from the Familial Hypercholesterolemia Variant Curation Expert Panel (FH VCEP) [15] were applied within the ACMG/AMP variant interpretation framework. FH VCEP provides gene-specific recommendations for the application of ACMG/AMP criteria rather than an independent classification system. For *APOB* and *PCSK9*, standard ACMG/AMP criteria were used due to the absence of gene-specific guidelines. The criteria (e.g., population frequency, computational prediction, and available literature and database evidence, including previously reported functional studies) were evaluated for each variant as applicable. When family data were available, segregation information was also used as supporting evidence. For downstream analysis, only pathogenic and likely pathogenic variants were considered. Variant classification was performed independently within this study. ClinVar annotations were used as supporting evidence but were not solely relied upon for final classification. Pathogenic and likely pathogenic variants were summarized and compared with ClinVar annotations (Table 2). Variants of uncertain significance (VUS), including those with conflicting interpretations in ClinVar, were reported separately without reclassification (S3 Table in S1 File).

**Table 2:** List of Pathogenic and Likely Pathogenic Variants Detected in Patients With FH.

№	Gene	Transcript (RefSeq)	HGVS (c.)	Exon/Intron Region	HGVS (p.)	rs ID	ClinVar ID	Pathogen. (ClinVar)	ACMG/AMP (this study)	Zygosity	N of Cases	Clin. Diagnosis
1	*LDLR*	NM_000527.5	c.1879G > A	Exon 13	p.Ala627Thr	rs879255066	252101	P/LP	P	Het	3	HoFH
2	*LDLR*	NM_000527.5	c.3G > A	Exon 1	p.Met1Ile	rs879254383	440536	LP	LP	Het	2	DLCN definite
3	*LDLR*	NM_000527.5	c.621C > T	Exon 4	p.Gly207=	rs121908044	3747	P	P	Het	1	DLCN probable
4	*LDLR*	NM_000527.5	c.1358 + 2T > C	Intron 9 (splice donor)	—	rs193922567	369862	P	P	Het	1	DLCN definite
5	*LDLR*	NM_000527.5	c.1187-10G > A	Intron 8 (splice region)	—	rs765696008	226349	P	P	Het	2	DLCN definite
6	*LDLR*	NM_000527.5	c.1747C > T	Exon 12	p.His583Tyr	rs730882109	200921	P	P	Het	1	DLCN definite
7	*LDLR*	NM_000527.5	c.622G > A	Exon 4	p.Glu208Lys	rs879254597	251328	P/LP	LP	Het	1	DLCN probable
8	*APOB*	NM_000384.3	c.10579C > T	Exon 26	p.Arg3527Trp	rs144467873	40223	P/LP	P	Het	1	DLCN possible
9	*APOB*	NM_000384.3	c.10580G > A	Exon 26	p.Arg3527Gln	rs5742904	17890	P/LP	LP	Het	1	DLCN definite
10	*LDLR*	NM_000527.5	c.418_426del	Exon 4	p.Glu140_Ser142del	—	not reported in ClinVar	—	LP	Het	3	HoFH

Notes: (P) pathogenic, (LP) likely pathogenic variant.

### 2.5. Statistical analysis

Statistical analyses were performed using IBM SPSS Statistics, version 29.0 (IBM Corp., Armonk, NY, USA). Descriptive data are presented as mean ± standard deviation (SD), or as median (Me) and interquartile range (25th–75th percentiles), as appropriate. The distribution of continuous variables was assessed using the Shapiro–Wilk and Kolmogorov–Smirnov tests. For normally distributed variables, Student’s t-test was used for between-group comparisons. When variables were not normally distributed, nonparametric tests were applied: the Wilcoxon signed-rank test for comparisons of two related/paired samples or repeated measurements, and the Mann–Whitney U test for comparisons between two independent groups. Differences in categorical variables were assessed using the chi-square (χ²) test, with Fisher’s exact test applied for small sample sizes. A two-sided p value <0.05 was considered statistically significant.

For the diagnostic accuracy analysis of the Dutch Lipid Clinic Network (DLCN) criteria, the presence of at least one pathogenic/likely pathogenic (P/LP) variant in FH genes (*LDLR*, *APOB*, *PCSK9*, *LDLRAP1*) was used as the reference standard for monogenic FH. The main index test was the DLCN category, and we also evaluated the prespecified binary threshold of DLCN >8 points versus ≤8 points, because this cut-off is embedded in the DLCN algorithm and corresponds to “definite FH”, a commonly used clinical decision point for prioritising patients for genetic testing. In this study, genetic testing was performed across DLCN strata by design, and lower DLCN categories were not excluded. Positive predictive value (PPV) was calculated for each DLCN category, and sensitivity, specificity, PPV, and negative predictive value (NPV) were calculated for the DLCN >8 threshold. Two-sided 95% confidence intervals (CIs) for proportions were calculated using the Wilson method.

## 3. Results

In the comparative analysis of the study groups (Table 1), patients with DLCN-predicted heterozygous familial hypercholesterolemia (HeFH) (Group II) were, on average, 5.7 years younger than the control group without FH (Group I). In Group II, a history of myocardial infarction was observed twice as often, percutaneous coronary intervention (PCI) 2.5 times as often, and coronary artery bypass grafting (CABG) four times as often. Although these differences were not statistically significant when considered individually, the cumulative burden of prior major adverse cardiovascular events (MACE) was twofold higher than in controls (p < 0.05). With respect to biochemical parameters, patients with HeFH had higher levels of total cholesterol (p < 0.05), low-density lipoprotein cholesterol (LDL-C) (p < 0.01), and apolipoprotein B (ApoB) (p < 0.01).

Because Group III included only three patients (an older brother and two sisters) with a phenotypic presentation of homozygous FH (HoFH), their characteristics in Table 1 are presented descriptively. Despite a mean age of 22.0 ± 3.6 years, CAD manifested at an early age: the index case (the brother) experienced an acute coronary syndrome (ACS) at 21 years and underwent CABG, while the youngest sister was hospitalized at 18 years due to unstable angina. Despite their young age, the median levels were markedly elevated: total cholesterol 750 mg/dL, LDL-C 705 mg/dL, and lipoprotein(a) [Lp(a)] 87 mg/dL (Table 1).

Regarding clinical FH signs (Table 1), tendon xanthomas and corneal arcus were observed only in the phenotypic FH groups (HeFH: 5/53 [9.4%] and 9/53 [17.0%], respectively; HoFH: 3/3 [100%] for both) and were absent in controls. Corneal arcus was significantly more frequent in HeFH than in controls (Fisher’s exact p = 0.009). Tendon xanthomas were observed in HeFH (5/53, 9.4%) and were absent in controls (0/39); this difference did not meet the conventional threshold for statistical significance (Fisher’s exact p = 0.070). A family history of premature CAD was common in both groups but was more frequent among HeFH patients than controls (49/53 [92.5%] vs 21/39 [53.8%], p < 0.001), consistent with clinical enrichment for FH.

Pathogenic and likely pathogenic variants identified in the cohort, along with their ACMG/AMP [15,14] classification and ClinVar annotations, are summarized in Table 2.

In the analysis of 95 patients, we identified 10 pathogenic (P) and likely pathogenic (LP) variants (Table 2), as well as 19 variants of uncertain significance (VUS) and variants with conflicting interpretations of pathogenicity (S3 Table in S1 File, Supplementary).

Among patients with heterozygous FH (HeFH), 8 pathogenic/likely pathogenic variants were detected, including 6 in *LDLR* and 2 in *APOB* (out of the 10 P/LP variants identified). All pathogenic variants were detected in index cases, except for one family in which both father and son were tested. This enabled cascade screening in nine index families to diagnose HeFH in first-degree relatives, including children, at an early stage.

Genetic testing confirmed the diagnosis in 7/15 (46.7%) patients with definite HeFH (DLCN), whereas confirmation was obtained in only 2/16 (12.5%) patients with probable HeFH and 1/22 (4.5%) patients with possible HeFH (Table 3). No P/LP variants were detected in the control group (DLCN 1–2 points). These findings support the highest diagnostic accuracy of the DLCN criteria at a score >8. At the same time, HeFH may still be missed in patients classified as probable or possible HeFH, underscoring the role of genetic testing as the diagnostic “gold standard.”

**Table 3:** Cross-tabulation of DLCN categories for HeFH and controls versus genetic confirmation (P/LP variants) and genetic confirmation rates (PPV) with 95% confidence intervals (including an overall FH [HeFH + HoFH] summary).

DLCN category/ study group	Genetic confirmation + (≥1 P/LP)	Genetic confirmation − (no P/LP)	Genetic confirmation rate (PPV), % (95% CI)
Overall FH (HeFH + HoFH), n = 56	13	43	23.2% (14.1–35.8)
Definite HeFH (>8 points), n = 15	7	8	46.7% (24.8–69.9)
Probable HeFH (6–8 points), n = 16	2	14	12.5% (3.5–36.0)
Possible HeFH (3–5 points), n = 22	1	21	4.5% (0.8–21.8)
Controls (DLCN 1–2 points), n = 39	0	39	0.0% (0.0–9.0)

Note: PPV (Positive Predictive Value) and 95% confidence intervals were calculated using the Wilson method. P/LP, pathogenic/likely pathogenic. VUS were not considered confirmatory for monogenic FH. The “Overall FH” row includes both HeFH and HoFH cases; DLCN categories apply to HeFH only.

Among HeFH patients, tendon xanthomas were more frequent in mutation-positive cases (3/10, 30.0%) than in mutation-negative cases (2/43, 4.7%; Fisher’s exact p = 0.041), whereas corneal arcus showed a borderline association (p = 0.053) (S5 Table in S1 File).

Genetic confirmation rates differed across DLCN categories (definite/probable/possible; χ² p = 0.0042; pairwise Fisher’s exact p = 0.0038 for definite vs possible; see S6 Table in S1 File).

In the three patients with a phenotypic presentation of homozygous FH (HoFH), two *LDLR* variants were identified. One, NM_000527.5:c.1879G > A (p.Ala627Thr), is a confirmed pathogenic variant listed in ClinVar; the second, NP_000518.1:p.Glu140_Ser142del, is a rare likely pathogenic variant affecting a critical receptor domain and was previously unreported. Family assessment showed definite HeFH in both parents based on the DLCN score; genetic testing in the mother confirmed the *LDLR* variant NM_000527.5:c.1879G > A (p.Ala627Thr), whereas the father was not genetically tested due to lack of written consent.

According to experts from the reference genetic laboratory Health in Code (Spain), consulted on this clinical case, the second *LDLR* variant (NP_000518.1:p.Glu140_Ser142del) represents an in-frame deletion of three amino acids (Glu140–Ser142). It affects a key region of the receptor responsible for LDL–LDLR binding. Variants of this type, typically classified as functional class 3 (impaired LDL binding) are a known cause of FH. The internal database of Health in Code includes at least 13 in-frame deletion/insertion/combined variants affecting the same functional/structural receptor domain in 80 carriers from 60 families diagnosed with FH. No polymorphisms have been described in this region, which further supports its functional importance. A similar molecular mechanism of impaired LDL binding has previously been clearly demonstrated for the nearby variant p.Pro105_Gly314delinsArg.

In the molecular genetics laboratory of the Republican Specialized Scientific and Practical Medical Center of Cardiology, a bioinformatic assessment of this deletion indicated that it involves a class A ligand-binding repeat domain (approximately 40 residues), characterized by six conserved cysteines and negatively charged residues that mediate binding to ApoB and ApoE. Loss of three amino acids, including a glutamic acid residue, may disrupt secondary structure (α-helix/β-sheet), alter local charge and topology, and impair binding to the lipoprotein particle or calcium (Table 4).

**Table 4:** ACMG/AMP Criteria–Based Classification of the NP_000518.1:p.Glu140_Ser142del Variant.

Criterion	Strength	Rationale
PM1	Moderate	Located in a critical, highly conserved functional region of the LDL receptor
PM2	Moderate	Absent from population databases (e.g., gnomAD)
PM4	Moderate	In-frame deletion within a functional domain
PP3	Supporting	In silico predictions and structural context support a deleterious effect

The combination of PM1, PM2, PM4, and PP3 criteria (Table 4) supports pathogenicity even in the absence of direct functional evidence. Considering the homozygous genotype and the family history—definite HeFH (DLCN) in both parents, including a maternally confirmed *LDLR* variant (NM_000527.5(*LDLR*):c.1879G > A (p.Ala627Thr))—and in line with the expert assessment from Health in Code, this variant supports classification as likely pathogenic (LP) according to ACMG/AMP criteria.

S3 Table in S1 File provides a list of 19 distinct variants of uncertain significance (VUS) and variants with conflicting interpretations of pathogenicity, observed in 28 of 95 patients. As shown in S4 Table in S1 File, VUS were observed in *APOB* (10 patients), *PCSK9* (14 patients), and *LDLRAP1* (4 patients). *APOB* and *PCSK9* VUS were observed 2.0–2.5-fold more frequently in patients with FH than in non-FH patients, warranting further evaluation of these variants.

## 4. Discussion

Pathogenic variants in *LDLR, APOB, PCSK9,* and *LDLRAP1* are the primary cause of familial hypercholesterolemia (FH) and, therefore, represent the diagnostic “gold standard.” The presence of a pathogenic genetic variant increases the risk of premature atherosclerotic cardiovascular disease (ASCVD) by 6–22-fold [16]. Because FH-causative variants differ substantially across populations, sequencing of the four candidate genes in different regions remains a pressing priority [17].

Early identification of FH enables timely diagnosis through targeted cascade genetic screening in first-degree relatives. Although individuals with FH inevitably incur lifelong healthcare costs, failure to establish the diagnosis in a timely manner can lead to exponentially higher expenditures due to premature coronary artery disease, surgical interventions, disability, and mortality [2].

In Uzbekistan, genetic evidence for FH remains limited and largely based on candidate-variant studies, and the diagnostic yield of NGS for monogenic FH in CAD patients is not well defined.

In this Discussion, we compared our findings with two contemporaneous limited studies conducted in Latvia [18] and Saint Petersburg [19]. These studies had some differences in sequencing strategy and analytical scope—ranging from whole-genome sequencing (WGS) in Latvia to targeted sequencing approaches in Uzbekistan and Saint Petersburg. In Saint Petersburg, a hybrid targeted panel for the genetic diagnosis of inherited dyslipidemias was used, including an expanded list of genes associated with monogenic dyslipidemias and a set of single-nucleotide variants linked to polygenic hypercholesterolemia and cardiovascular risk; such methodological differences should be considered when comparing diagnostic yield and variant spectra across cohorts. These cohorts were selected because they are recent FH sequencing studies reporting diagnostic yield and variant spectra using comparable clinical criteria, enabling contextual interpretation of our results across geographically relevant Eurasian populations.

At the initial stage of our study, pathogenic/likely pathogenic (P/LP) variants were identified in 10 of 53 patients with clinically suspected heterozygous FH (HeFH), yielding an overall genetic confirmation rate of 18.9% in this subgroup. This is comparable to the Latvian WGS-based study (20.9% of 163 clinically diagnosed HeFH patients) and lower than the recent Saint Petersburg cohort, where the diagnostic yield based on P/LP findings was approximately 35.2% after excluding variants of uncertain significance (VUS). Such differences are not unexpected and may reflect variation in patient selection (CAD-based recruitment vs lipid clinic–based cohorts), phenotypic severity, background population genetics, and analytical strategies (gene content, handling of VUS, and availability of complementary approaches such as polygenic risk assessment and/or copy-number variant detection).

The modest overall genetic confirmation rate in our cohort likely reflects the study design and clinical setting. We deliberately enrolled patients across the full DLCN spectrum (possible/probable/definite) to evaluate clinical stratification against genetic testing; therefore, a substantial proportion of participants—especially in the probable/possible range—may have polygenic hypercholesterolemia or mixed aetiologies rather than classical monogenic FH. In addition, we defined genetic confirmation strictly as the presence of P/LP variants (excluding VUS) and did not perform copy-number/structural variant analysis, which may further reduce detectable yield in panel-based sequencing studies [16,17]. Finally, secondary causes of hypercholesterolemia were assessed during routine clinical evaluation and were explicitly excluded (Methods 2.1).

Across DLCN categories, genetic confirmation was consistently highest in the definite subgroup, although the magnitude differed markedly between studies (66.7% in Saint Petersburg, 47.0% in Uzbekistan, and ~21% in Latvia). In the probable subgroup, Latvia demonstrated a higher genetic confirmation rate (~21%) than our Uzbek cohort (12.5%). These observations support the concept that the diagnostic performance of clinical scoring systems is context-dependent and may vary by population and clinical setting, particularly in the intermediate probability range where polygenic hypercholesterolemia and secondary contributors may be more prevalent.

Taken together, our results suggest that classical clinical FH signs (particularly tendon xanthomas) can enrich for mutation-positive HeFH, but their sensitivity is limited and they do not replace genetic testing. Therefore, combining clinical stratification (e.g., DLCN) with targeted sequencing remains the most informative approach for identifying monogenic FH in CAD-based cohorts.

These findings support the use of the DLCN >8 (“definite FH”) threshold as the most efficient clinical stratum for genetic confirmation in this CAD-based cohort, while also highlighting that monogenic FH can still be present in the probable/possible range. The “Overall FH (HeFH+HoFH)” row provides a robustness summary of genetic confirmation across phenotypic FH in our enriched sample and should be interpreted in the context of the study design and sample size.

In our cohort, the P/LP spectrum was dominated by *LDLR*, accounting for 8 of 10 unique P/LP variants and 8 of 10 genetically confirmed HeFH cases, whereas *APOB* accounted for 2 of 10 unique variants and cases. Notably, both *APOB* P/LP variants involved codon 3527 (p.Arg3527Trp and p.Arg3527Gln), a well-recognized hotspot for familial defective ApoB-100, which may contribute to recurrent detection across different Eurasian/European cohorts. Variant overlap across studies was limited: only *LDLR* rs765696008 and *APOB* rs5742904 overlapped between our cohort and Saint Petersburg, and only *APOB* rs5742904 overlapped with Latvia. The *APOB* rs5742904 variant was detected in one index case in our cohort, compared with six cases in Saint Petersburg and nine cases in Latvia. Overall, these findings highlight population-specific FH genetic architectures and underscore the value of region-specific sequencing efforts to optimize genetic confirmation, refine risk stratification in patients with intermediate DLCN scores, and support cascade screening strategies.

Consistent with our findings, a recent comparative study of two national cohorts of patients with a clinical diagnosis of FH also reported pronounced between-population genetic heterogeneity, with only limited overlap in the detected variants [20].

Together, these observations highlight that FH genetic architecture is strongly population-specific and underscore the need for broader sequencing efforts across diverse populations to improve genotype–phenotype correlations and support diagnostic and cascade screening strategies.

Future directions. In larger, regionally representative Uzbek cohorts, machine-learning models that combine DLCN components, lipid measures, and genetic findings could be developed to provide region- and population-adapted risk stratification and to refine classification in the intermediate DLCN range. Such models should be trained with cross-validation and tested in an independent cohort to minimise overfitting.

Genetic testing may be particularly informative in patients with definite or probable FH according to clinical criteria, where it can confirm the diagnosis and support cascade screening. However, routine use in all patients with coronary artery disease may not be justified, particularly in resource-limited settings, given the cost of next-generation sequencing (NGS). A more targeted approach based on clinical preselection may therefore be more appropriate.

In this context, our study represents a first step toward mapping the FH variant spectrum in Uzbekistan and Central Asia and contributes to the larger global effort to capture the full breadth of FH genetic diversity.

To our knowledge, prior genetic evidence on FH in Uzbekistan has been scarce and largely limited to candidate variants/polymorphisms (e.g., PCR-RFLP genotyping of the *PCSK9* E670G polymorphism [rs505151], including *LDLR* mutations and an *APOB* mutation reported in a CAD + HeFH cohort) [21,22] rather than systematic NGS-based sequencing of FH genes, underscoring the novelty of our approach.

### 4.1. Limitations

This was a single-center study with an enriched, non-random (convenience) sample of CAD patients selected by DLCN strata and a separate low-DLCN control group, which may limit generalisability and could bias some accuracy estimates compared with an unselected population. The sample size was modest, leading to wide confidence intervals. Genetic confirmation was based on P/LP variants in *LDLR*, *APOB*, *PCSK9*, and *LDLRAP1*; large structural variants (e.g., CNVs) and additional rare genes associated with monogenic FH or FH-like phenotypes, such as *APOE*, *LIPA*, and other less frequent contributors, were not evaluated, potentially underestimating the genetic yield. Although such rare contributors are estimated to account for only a small proportion of FH cases, approximately 2–4% at most, their inclusion should be considered in future studies, particularly in larger cohorts and when broader genetic testing becomes feasible. Variants of uncertain significance were not considered confirmatory, and some misclassification remains possible as variant interpretation evolves. We did not develop machine-learning models because the modest sample size and enriched sampling design would likely lead to overfitting; this should be addressed in larger, regionally representative cohorts. Detailed lipid-lowering treatment regimens, adherence, and longitudinal outcomes (including therapy effects on MACE risk) were not analysed in this diagnostic-yield report; these data are being collected prospectively as part of the ongoing project and will be reported in a subsequent follow-up study. Polygenic risk scores (PGS) were not assessed in this study; future work in larger, regionally representative Uzbek cohorts should incorporate PGS to better distinguish monogenic FH from polygenic hypercholesterolemia, particularly in the probable/possible DLCN range. Finally, the index test (DLCN scoring) and reference standard (NGS) were not formally blinded, which may introduce information bias.

## 5. Conclusion

Based on the first NGS-based study in Uzbekistan and Central Asia investigating the genetic features of Familial hypercholesterolemia in patients with coronary artery disease (CAD), 10 pathogenic/likely pathogenic variants and 19 variants of uncertain significance (VUS), including variants with conflicting interpretations of pathogenicity, were identified. Genetic testing confirmed the diagnosis in 7/15 (46.7%) patients with definite heterozygous FH (HeFH; DLCN), whereas confirmation was obtained in only 2/16 (12.5%) patients with probable HeFH and 1/22 (4.5%) patients with possible HeFH. These findings support genetic testing as the diagnostic “gold standard” for HeFH and confirm the validity of the DLCN score when >8 points, while not excluding FH in patients with lower scores.

In the three patients with CAD and phenotypic presentation of homozygous FH included in this study, two *LDLR* variants were detected on different alleles at the same locus, consistent with a diagnosis of compound heterozygosity. The second variant (p.Glu140_Ser142del) is reported here for the first time and based on bioinformatic analysis, is considered likely pathogenic.

## Supporting information

S1 File Supporting information file containing S1 Text, S2 Text, and S3–S7 Tables. (DOCX)

S1 Checklist STARD 2015 checklist. (DOCX)
