# Supplementary material for: Genetic yield of next-generation sequencing for detecting monogenic familial hypercholesterolemia in uzbek patients with coronary artery disease
Source: PLoS One. 2026 Jul 9;21(7):e0353401. doi: 10.1371/journal.pone.0353401 (PMC13349120; doi:10.1371/journal.pone.0353401)
Supplement: S1 File — (DOCX) [file pone.0353401.s001.docx]

**S1 Text.** **Instrumental diagnostic procedures used for CAD confirmation and eligibility assessment**

1. 12-lead electrocardiogram (ECG);

2. Echocardiography;

3. Carotid ultrasound;

4. 24-hour Holter monitoring;

5. Treadmill test;

6. CT/coronary angiography as indicated.

**S2 Text. Online resources and software documentation**

OR1 QIAGEN. QIAseq Targeted DNA Pro Handbook. July 2024.

OR2 QIAGEN Digital Insights. CLC Genomics Workbench User Manual.

OR3 QIAGEN Digital Insights. Biomedical Genomics Analysis User Manual.

OR4 QIAGEN. QIAGEN Clinical Insight (QCI) Interpret online platform resources

OR5 Alieva R, Shek A, Bahachova A, Fozilov K, Abdullaeva G, Abdullaev A, et al. Targeted DNA Sequencing of *LDLR*, *APOB*, *PCSK9*, and *LDLRAP1* Using QIAseq DNA Pro Custom Panel and Illumina MiSeq. protocols.io; 2026. Available from: <https://dx.doi.org/10.17504/protocols.io.bp2l6jqbkvqe/v1>

OR6 Instrumental diagnostic procedures used for CAD confirmation and eligibility assessment (ECG, echocardiography, carotid ultrasound, Holter monitoring, treadmill test, and CT/coronary angiography as indicated).

**S3 Table. List of variants of uncertain significance (VUS) and variants with conflicting interpretations of pathogenicity**

| № | Gene | Variant, Nomenclature | rs ID | ClinVar ID | Pathogenicity (ClinVar) | Zygosity | Number of Cases |
| --- | --- | --- | --- | --- | --- | --- | --- |
| 1 | *APOB* | NM_000384.3(*APOB*):c.1352+60C>A | rs12714224 | 265879 | VUS | Heterozyg. | 1 |
| 2 | *APOB* | NM_000384.3(*APOB*):c.640C>T (p.Arg214Cys)  g.21260025G>A. | rs763352655 | 1753371 | VUS/LB  (conflicting pathogenicity) | Heterozyg. | 1 |
| 3 | *APOB* | NM_000384.3(*APOB*):c.*179C>T | rs142151703 | 334060 | VUS/LB  (conflicting pathogenicity) | Heterozyg. | 2 |
| 4 | *APOB* | NM_000384.3(*APOB*):c.7615G>A (p.Val2539Ile) | rs148170480 | 334125 | VUS/LB  (conflicting pathogenicity) | Heterozyg. | 1 |
| 5 | *APOB* | NM_000384.3(*APOB*):c.9477G>A (p.Lys3159=) | rs13306196 | 334113 | VUS/LB  (conflicting pathogenicity) | Homozyg. | 1 |
| 6 | *APOB* | NM_000384.3(*APOB*):c.2706C>T (p.Asn902=) | rs1801700 | 255981 | VUS/B  (conflicting pathogenicity) | Heterozyg. | 1 |
| 7 | *APOB* | NM_000384.3(*APOB*):c.1594C>T (p.Arg532Trp) | rs13306194 | 255979 | VUS/B  (conflicting pathogenicity) | Homozyg. | 1 |
| 8 | *APOB* | NM_000384.3(*APOB*):c.13441G>A (p.Ala4481Thr) | rs1801695 | 128419 | VUS/B  (conflicting pathogenicity) | Heterozyg. | 1 |
| 9 | *APOB* | NM_000384.3(*APOB*):c.4111G>A (p.Ala1371Thr) | rs780170292 | 548055 | VUS/LB  (conflicting pathogenicity) | Heterozyg. | 1 |
| 10 | *PCSK9* | NM_174936.4(*PCSK9*):c.158C>G (p.Ala53Gly) | rs11583680 | 440708 | VUS | Heterozyg. | 3 |
| 11 | *PCSK9* | NM_174936.4(*PCSK9*):c.657+114del  g55052525del | [rs397735050](https://www.ncbi.nlm.nih.gov/snp/rs397735050) | 265928 | VUS | Heterozyg. | 1 |
| 12 | *PCSK9* | NM_174936.4(*PCSK9*):c.-237T>G | rs886046427 | 297686 | VUS | Heterozyg. | 1 |
| 13 | *PCSK9* | NM_174936.4(*PCSK9*):c.706G>A (p.Gly236Ser) | rs149489325 | 403289 | VUS | Heterozyg. | 2 |
| 14 | *PCSK9* | NM_174936.4(*PCSK9*):c.1026A>G (p.Gln342=) | rs509504 | 262899 | VUS/B  (conflicting pathogenicity) | Homozyg. | 1 |
| 15 | *PCSK9* | NM_174936.4(*PCSK9*):c.1026A>G (p.Gln342=) | rs509504 | 262899 | VUS/B  (conflicting pathogenicity) | Heterozyg. | 2 |
| 16 | *PCSK9* | NM_174936.4(*PCSK9*):c.720C>T (p.Gly240=) | rs41297883 | 242028 | VUS/B  (conflicting pathogenicity) | Heterozyg. | 1 |
| 17 | *PCSK9* | NM_174936.4(*PCSK9*):c.1864-13C>T | rs147470944 | 548106 | VUS/LB (conflicting pathogenicity) | Heterozyg. | 3 |
| 18 | *LDLRAP1* | NM_015627.3(*LDLRAP1*):c.362C>T (p.Ala121Val) | rs763142541 | 2448693 | VUS | Heterozyg. | 2 |
| 19 | *LDLRAP1* | NM_015627.3(*LDLRAP1*):c.712C>T (p.Arg238Trp)  g.25563756C>T | rs41291058 | 296986 | VUS/LB  (conflicting pathogenicity) | Heterozyg. | 2 |

Abbreviations: (VUS) variant of uncertain significance, B – benign, LB – possibly benign

**S4 Table. Distribution of patients carrying variants of uncertain significance (VUS) and VUS with conflicting interpretations of pathogenicity in *APOB*, *PCSK9*, and *LDLRAP1* across study groups**

| Group | ***APOB***, n (%) | ***PCSK9***, n (%) | ***LDLRAP1***, n (%) | **Patients with ≥1 VUS, n** |
| --- | --- | --- | --- | --- |
| HoFH | – | 2 (14) | – | 2 |
| HeFH | 7 (70) | 8 (57) | 2 (50) | 17 |
| non-FH | 3 (30) | 4 (29) | 2 (50) | 9 |
| **Total (patients), n** | **10** | **14** | **4** | **28** |

Data are presented as n (%), where percentages are calculated within each gene (column total). The last column shows the number of patients with ≥1 VUS/VUS (conflicting) in any.

**S5 Table. Clinical FH signs in HeFH according to mutation status**

Association of tendon xanthomas, corneal arcus, and family history of premature CAD with P/LP variant–positive status among HeFH patients.

| Parameter | HeFH mutation+ (n=10) | HeFH mutation− (n=43) | OR | P value |
| --- | --- | --- | --- | --- |
| Tendon xanthomas | 3 (30.0%) | 2 (4.7%) | 8.79 | 0.041 |
| Corneal arcus | 4 (40.0%) | 5 (11.6%) | 5.07 | 0.053 |

P values: Fisher’s exact test. OR: odds ratio (Fisher).

Note: ‘mutation+’ indicates ≥1 pathogenic/likely pathogenic (P/LP) variant; controls were all mutation− by definition. Family history of premature CAD was not analysed in this table because no mutation-positive HeFH cases without a family history of premature CAD were observed (zero-cell problem for odds ratio estimation).

**S6 Table. Statistical comparison of genetic confirmation yield by DLCN category (HeFH)**

| Comparison | Test | OR | P value |
| --- | --- | --- | --- |
| Overall across DLCN categories (definite/probable/possible) | χ² | — | 0.0042 |
| Definite vs Possible | Fisher | 18.38 | 0.0038 |
| Definite vs Probable | Fisher | 6.12 | 0.0538 |
| Probable vs Possible | Fisher | 3.00 | 0.5619 |

Note: Genetic confirmation defined as presence of ≥1 pathogenic/likely pathogenic (P/LP) variant.

P values: χ² test across categories and Fisher’s exact tests for pairwise comparisons. OR: odds ratio (Fisher).

Pairwise p values are unadjusted (reported descriptively). Underlying counts and PPV with 95% CIs are reported in Table 3 of the main manuscript.

**S7 Table. Amplicon design and primer coordinates for the custom QIAseq DNA Pro panel covering *LDLR*, *APOB*, *PCSK9*, and *LDLRAP1***

| **Chromo-some** | **Amplicon start** | **Amplicon end** | **Gene** | **Amplicon strand** | **Primer start** | **Primer**  **end** |
| --- | --- | --- | --- | --- | --- | --- |
| chr1 | 25543631 | 25543881 | *LDLRAP1* | + | 25543631 | 25543655 |
| chr1 | 25543694 | 25543944 | *LDLRAP1* | + | 25543694 | 25543714 |
| chr1 | 25553830 | 25554080 | *LDLRAP1* | + | 25553830 | 25553856 |
| chr1 | 25553875 | 25554125 | *LDLRAP1* | - | 25554101 | 25554125 |
| chr1 | 25554776 | 25555026 | *LDLRAP1* | + | 25554776 | 25554807 |
| chr1 | 25554803 | 25555053 | *LDLRAP1* | - | 25555023 | 25555053 |
| chr1 | 25557168 | 25557418 | *LDLRAP1* | + | 25557168 | 25557195 |
| chr1 | 25557025 | 25557275 | *LDLRAP1* | - | 25557252 | 25557275 |
| chr1 | 25562498 | 25562748 | *LDLRAP1* | - | 25562719 | 25562748 |
| chr1 | 25562553 | 25562803 | *LDLRAP1* | - | 25562780 | 25562803 |
| chr1 | 25562979 | 25563229 | *LDLRAP1* | + | 25562979 | 25563002 |
| chr1 | 25563062 | 25563312 | *LDLRAP1* | + | 25563062 | 25563091 |
| chr1 | 25563502 | 25563752 | *LDLRAP1* | - | 25563727 | 25563752 |
| chr1 | 25563588 | 25563838 | *LDLRAP1* | - | 25563813 | 25563838 |
| chr1 | 25565065 | 25565315 | *LDLRAP1* | + | 25565065 | 25565093 |
| chr1 | 25565064 | 25565314 | *LDLRAP1* | - | 25565283 | 25565314 |
| chr1 | 25566707 | 25566957 | *LDLRAP1* | - | 25566934 | 25566957 |
| chr1 | 25566797 | 25567047 | *LDLRAP1* | - | 25567022 | 25567047 |
| chr1 | 55039791 | 55040041 | *PCSK9* | + | 55039791 | 55039815 |
| chr1 | 55039775 | 55040025 | *PCSK9* | - | 55040004 | 55040025 |
| chr1 | 55039898 | 55040148 | *PCSK9* | - | 55040120 | 55040148 |
| chr1 | 55043791 | 55044041 | *PCSK9* | + | 55043791 | 55043820 |
| chr1 | 55043777 | 55044027 | *PCSK9* | - | 55044003 | 55044027 |
| chr1 | 55043861 | 55044111 | *PCSK9* | - | 55044080 | 55044111 |
| chr1 | 55046470 | 55046720 | *PCSK9* | + | 55046470 | 55046497 |
| chr1 | 55046460 | 55046710 | *PCSK9* | - | 55046686 | 55046710 |
| chr1 | 55052211 | 55052461 | *PCSK9* | + | 55052211 | 55052249 |
| chr1 | 55052297 | 55052547 | *PCSK9* | + | 55052297 | 55052329 |
| chr1 | 55052553 | 55052803 | *PCSK9* | + | 55052553 | 55052578 |
| chr1 | 55052587 | 55052837 | *PCSK9* | - | 55052813 | 55052837 |
| chr1 | 55055898 | 55056148 | *PCSK9* | + | 55055898 | 55055930 |
| chr1 | 55055989 | 55056239 | *PCSK9* | + | 55055989 | 55056016 |
| chr1 | 55056065 | 55056315 | *PCSK9* | + | 55056065 | 55056087 |
| chr1 | 55057219 | 55057469 | *PCSK9* | + | 55057219 | 55057248 |
| chr1 | 55057246 | 55057496 | *PCSK9* | - | 55057467 | 55057496 |
| chr1 | 55057316 | 55057566 | *PCSK9* | - | 55057541 | 55057566 |
| chr1 | 55058082 | 55058332 | *PCSK9* | + | 55058082 | 55058110 |
| chr1 | 55057893 | 55058143 | *PCSK9* | - | 55058114 | 55058143 |
| chr1 | 55058409 | 55058659 | *PCSK9* | + | 55058409 | 55058442 |
| chr1 | 55058532 | 55058782 | *PCSK9* | + | 55058532 | 55058554 |
| chr1 | 55059425 | 55059675 | *PCSK9* | + | 55059425 | 55059461 |
| chr1 | 55059573 | 55059723 | *PCSK9* | - | 55059695 | 55059723 |
| chr1 | 55061264 | 55061514 | *PCSK9* | + | 55061264 | 55061295 |
| chr1 | 55061290 | 55061540 | *PCSK9* | - | 55061512 | 55061540 |
| chr1 | 55061369 | 55061619 | *PCSK9* | - | 55061593 | 55061619 |
| chr1 | 55063282 | 55063532 | *PCSK9* | + | 55063282 | 55063311 |
| chr1 | 55063288 | 55063538 | *PCSK9* | - | 55063516 | 55063538 |
| chr1 | 55063408 | 55063658 | *PCSK9* | - | 55063628 | 55063658 |
| chr19 | 11089488 | 11089738 | *LDLR* | + | 11089488 | 11089513 |
| chr19 | 11100161 | 11100411 | *LDLR* | + | 11100161 | 11100190 |
| chr19 | 11100256 | 11100506 | *LDLR* | + | 11100256 | 11100285 |
| chr19 | 11102612 | 11102862 | *LDLR* | + | 11102612 | 11102640 |
| chr19 | 11102614 | 11102864 | *LDLR* | - | 11102837 | 11102864 |
| chr19 | 11105137 | 11105387 | *LDLR* | + | 11105137 | 11105161 |
| chr19 | 11105252 | 11105502 | *LDLR* | + | 11105252 | 11105277 |
| chr19 | 11105242 | 11105492 | *LDLR* | - | 11105465 | 11105492 |
| chr19 | 11105356 | 11105606 | *LDLR* | - | 11105576 | 11105606 |
| chr19 | 11105444 | 11105694 | *LDLR* | - | 11105667 | 11105694 |
| chr19 | 11106500 | 11106750 | *LDLR* | + | 11106500 | 11106533 |
| chr19 | 11106581 | 11106831 | *LDLR* | + | 11106581 | 11106608 |
| chr19 | 11107289 | 11107539 | *LDLR* | + | 11107289 | 11107331 |
| chr19 | 11107337 | 11107587 | *LDLR* | - | 11107558 | 11107587 |
| chr19 | 11110582 | 11110832 | *LDLR* | + | 11110582 | 11110604 |
| chr19 | 11110578 | 11110828 | *LDLR* | - | 11110805 | 11110828 |
| chr19 | 11111447 | 11111697 | *LDLR* | + | 11111447 | 11111471 |
| chr19 | 11111487 | 11111737 | *LDLR* | - | 11111704 | 11111737 |
| chr19 | 11113215 | 11113465 | *LDLR*;*MIR6886* | + | 11113215 | 11113240 |
| chr19 | 11113336 | 11113586 | *LDLR*;*MIR6886* | + | 11113336 | 11113359 |
| chr19 | 11113402 | 11113652 | *LDLR*;*MIR6886* | - | 11113624 | 11113652 |
| chr19 | 11113474 | 11113724 | *LDLR*;*MIR6886* | - | 11113694 | 11113724 |
| chr19 | 11113592 | 11113842 | *LDLR*;*MIR6886* | - | 11113817 | 11113842 |
| chr19 | 11116007 | 11116257 | *LDLR* | + | 11116007 | 11116037 |
| chr19 | 11116093 | 11116343 | *LDLR* | + | 11116093 | 11116122 |
| chr19 | 11116717 | 11116967 | *LDLR* | - | 11116937 | 11116967 |
| chr19 | 11116821 | 11117071 | *LDLR* | - | 11117035 | 11117071 |
| chr19 | 11120031 | 11120281 | *LDLR* | + | 11120031 | 11120059 |
| chr19 | 11120046 | 11120296 | *LDLR* | - | 11120264 | 11120296 |
| chr19 | 11120313 | 11120563 | *LDLR* | + | 11120313 | 11120344 |
| chr19 | 11120363 | 11120613 | *LDLR* | - | 11120582 | 11120613 |
| chr19 | 11123108 | 11123358 | *LDLR* | + | 11123108 | 11123131 |
| chr19 | 11123152 | 11123402 | *LDLR* | - | 11123380 | 11123402 |
| chr19 | 11127956 | 11128206 | *LDLR* | + | 11127956 | 11127982 |
| chr19 | 11129378 | 11129628 | *LDLR* | + | 11129378 | 11129413 |
| chr19 | 11129547 | 11129797 | *LDLR* | + | 11129547 | 11129579 |
| chr19 | 11129380 | 11129630 | *LDLR* | - | 11129600 | 11129630 |
| chr19 | 11131281 | 11131531 | *LDLR* | + | 11131281 | 11131309 |
| chr19 | 11131117 | 11131367 | *LDLR* | - | 11131344 | 11131367 |
| chr19 | 11131331 | 11131581 | *LDLR* | - | 11131549 | 11131581 |
| chr2 | 21001653 | 21001903 | *APOB* | + | 21001653 | 21001689 |
| chr2 | 21001762 | 21002012 | *APOB* | + | 21001762 | 21001794 |
| chr2 | 21001800 | 21002050 | *APOB* | - | 21002017 | 21002050 |
| chr2 | 21001914 | 21002164 | *APOB* | - | 21002126 | 21002164 |
| chr2 | 21002171 | 21002421 | *APOB* | + | 21002171 | 21002208 |
| chr2 | 21002023 | 21002273 | *APOB* | - | 21002235 | 21002273 |
| chr2 | 21002289 | 21002539 | *APOB* | + | 21002289 | 21002319 |
| chr2 | 21002499 | 21002749 | *APOB* | + | 21002499 | 21002537 |
| chr2 | 21002332 | 21002582 | *APOB* | - | 21002547 | 21002582 |
| chr2 | 21002549 | 21002799 | *APOB* | - | 21002765 | 21002799 |
| chr2 | 21002819 | 21003069 | *APOB* | + | 21002819 | 21002854 |
| chr2 | 21002662 | 21002912 | *APOB* | - | 21002882 | 21002912 |
| chr2 | 21003024 | 21003274 | *APOB* | + | 21003024 | 21003058 |
| chr2 | 21002867 | 21003117 | *APOB* | - | 21003085 | 21003117 |
| chr2 | 21003141 | 21003391 | *APOB* | + | 21003141 | 21003172 |
| chr2 | 21003230 | 21003480 | *APOB* | + | 21003230 | 21003263 |
| chr2 | 21004212 | 21004462 | *APOB* | + | 21004212 | 21004237 |
| chr2 | 21004259 | 21004509 | *APOB* | - | 21004474 | 21004509 |
| chr2 | 21004426 | 21004676 | *APOB* | - | 21004643 | 21004676 |
| chr2 | 21004493 | 21004743 | *APOB* | - | 21004703 | 21004743 |
| chr2 | 21005138 | 21005388 | *APOB* | + | 21005138 | 21005174 |
| chr2 | 21004959 | 21005209 | *APOB* | - | 21005178 | 21005209 |
| chr2 | 21005250 | 21005500 | *APOB* | + | 21005250 | 21005285 |
| chr2 | 21005360 | 21005610 | *APOB* | + | 21005360 | 21005390 |
| chr2 | 21005471 | 21005721 | *APOB* | + | 21005471 | 21005507 |
| chr2 | 21005578 | 21005828 | *APOB* | + | 21005578 | 21005611 |
| chr2 | 21005689 | 21005939 | *APOB* | + | 21005689 | 21005725 |
| chr2 | 21005799 | 21006049 | *APOB* | + | 21005799 | 21005835 |
| chr2 | 21005844 | 21006094 | *APOB* | - | 21006065 | 21006094 |
| chr2 | 21005961 | 21006211 | *APOB* | - | 21006182 | 21006211 |
| chr2 | 21006231 | 21006381 | *APOB* | + | 21006231 | 21006267 |
| chr2 | 21006178 | 21006328 | *APOB* | - | 21006296 | 21006328 |
| chr2 | 21006348 | 21006598 | *APOB* | + | 21006348 | 21006379 |
| chr2 | 21006398 | 21006648 | *APOB* | - | 21006615 | 21006648 |
| chr2 | 21006689 | 21006939 | *APOB* | + | 21006689 | 21006720 |
| chr2 | 21006510 | 21006760 | *APOB* | - | 21006726 | 21006760 |
| chr2 | 21006739 | 21006989 | *APOB* | - | 21006954 | 21006989 |
| chr2 | 21007033 | 21007283 | *APOB* | + | 21007033 | 21007061 |
| chr2 | 21006852 | 21007102 | *APOB* | - | 21007067 | 21007102 |
| chr2 | 21007145 | 21007395 | *APOB* | + | 21007145 | 21007181 |
| chr2 | 21007259 | 21007509 | *APOB* | + | 21007259 | 21007293 |
| chr2 | 21007364 | 21007614 | *APOB* | + | 21007364 | 21007398 |
| chr2 | 21007592 | 21007842 | *APOB* | + | 21007592 | 21007620 |
| chr2 | 21007414 | 21007664 | *APOB* | - | 21007629 | 21007664 |
| chr2 | 21007707 | 21007957 | *APOB* | + | 21007707 | 21007739 |
| chr2 | 21007829 | 21008079 | *APOB* | + | 21007829 | 21007855 |
| chr2 | 21007872 | 21008122 | *APOB* | - | 21008095 | 21008122 |
| chr2 | 21007990 | 21008240 | *APOB* | - | 21008205 | 21008240 |
| chr2 | 21008283 | 21008533 | *APOB* | + | 21008283 | 21008315 |
| chr2 | 21008203 | 21008353 | *APOB* | - | 21008319 | 21008353 |
| chr2 | 21008393 | 21008643 | *APOB* | + | 21008393 | 21008424 |
| chr2 | 21008513 | 21008763 | *APOB* | + | 21008513 | 21008541 |
| chr2 | 21008563 | 21008813 | *APOB* | - | 21008782 | 21008813 |
| chr2 | 21008680 | 21008930 | *APOB* | - | 21008897 | 21008930 |
| chr2 | 21008794 | 21009044 | *APOB* | - | 21009009 | 21009044 |
| chr2 | 21009091 | 21009341 | *APOB* | + | 21009091 | 21009121 |
| chr2 | 21008907 | 21009157 | *APOB* | - | 21009125 | 21009157 |
| chr2 | 21009141 | 21009391 | *APOB* | - | 21009357 | 21009391 |
| chr2 | 21009435 | 21009685 | *APOB* | + | 21009435 | 21009465 |
| chr2 | 21009252 | 21009502 | *APOB* | - | 21009469 | 21009502 |
| chr2 | 21009544 | 21009794 | *APOB* | + | 21009544 | 21009582 |
| chr2 | 21009657 | 21009907 | *APOB* | + | 21009657 | 21009692 |
| chr2 | 21009768 | 21010018 | *APOB* | + | 21009768 | 21009804 |
| chr2 | 21009990 | 21010240 | *APOB* | + | 21009990 | 21010026 |
| chr2 | 21009818 | 21010068 | *APOB* | - | 21010031 | 21010068 |
| chr2 | 21010102 | 21010352 | *APOB* | + | 21010102 | 21010138 |
| chr2 | 21010131 | 21010381 | *APOB* | - | 21010340 | 21010381 |
| chr2 | 21010235 | 21010485 | *APOB* | - | 21010453 | 21010485 |
| chr2 | 21010520 | 21010770 | *APOB* | + | 21010520 | 21010553 |
| chr2 | 21010342 | 21010592 | *APOB* | - | 21010557 | 21010592 |
| chr2 | 21010570 | 21010820 | *APOB* | - | 21010786 | 21010820 |
| chr2 | 21010874 | 21011124 | *APOB* | + | 21010874 | 21010904 |
| chr2 | 21010684 | 21010934 | *APOB* | - | 21010908 | 21010934 |
| chr2 | 21010924 | 21011174 | *APOB* | - | 21011147 | 21011174 |
| chr2 | 21011204 | 21011454 | *APOB* | + | 21011204 | 21011240 |
| chr2 | 21011044 | 21011294 | *APOB* | - | 21011267 | 21011294 |
| chr2 | 21011322 | 21011572 | *APOB* | + | 21011322 | 21011352 |
| chr2 | 21011432 | 21011682 | *APOB* | + | 21011432 | 21011466 |
| chr2 | 21011538 | 21011788 | *APOB* | + | 21011538 | 21011574 |
| chr2 | 21011653 | 21011903 | *APOB* | + | 21011653 | 21011686 |
| chr2 | 21011761 | 21012011 | *APOB* | + | 21011761 | 21011794 |
| chr2 | 21011811 | 21012061 | *APOB* | - | 21012030 | 21012061 |
| chr2 | 21011928 | 21012178 | *APOB* | - | 21012142 | 21012178 |
| chr2 | 21012037 | 21012287 | *APOB* | - | 21012257 | 21012287 |
| chr2 | 21012155 | 21012405 | *APOB* | - | 21012374 | 21012405 |
| chr2 | 21012269 | 21012519 | *APOB* | - | 21012483 | 21012519 |
| chr2 | 21012360 | 21012610 | *APOB* | - | 21012579 | 21012610 |
| chr2 | 21012477 | 21012727 | *APOB* | - | 21012688 | 21012727 |
| chr2 | 21013098 | 21013348 | *APOB* | + | 21013098 | 21013127 |
| chr2 | 21013135 | 21013385 | *APOB* | - | 21013353 | 21013385 |
| chr2 | 21013250 | 21013500 | *APOB* | - | 21013465 | 21013500 |
| chr2 | 21013352 | 21013602 | *APOB* | - | 21013570 | 21013602 |
| chr2 | 21014312 | 21014562 | *APOB* | - | 21014530 | 21014562 |
| chr2 | 21014411 | 21014661 | *APOB* | - | 21014628 | 21014661 |
| chr2 | 21014947 | 21015197 | *APOB* | - | 21015165 | 21015197 |
| chr2 | 21015063 | 21015313 | *APOB* | - | 21015282 | 21015313 |
| chr2 | 21015313 | 21015563 | *APOB* | + | 21015313 | 21015343 |
| chr2 | 21015357 | 21015607 | *APOB* | - | 21015571 | 21015607 |
| chr2 | 21016355 | 21016605 | *APOB* | + | 21016355 | 21016393 |
| chr2 | 21016392 | 21016642 | *APOB* | - | 21016606 | 21016642 |
| chr2 | 21016455 | 21016705 | *APOB* | - | 21016672 | 21016705 |
| chr2 | 21018914 | 21019164 | *APOB* | + | 21018914 | 21018950 |
| chr2 | 21018928 | 21019178 | *APOB* | - | 21019141 | 21019178 |
| chr2 | 21019611 | 21019861 | *APOB* | + | 21019611 | 21019640 |
| chr2 | 21019672 | 21019922 | *APOB* | + | 21019672 | 21019701 |
| chr2 | 21019714 | 21019964 | *APOB* | - | 21019928 | 21019964 |
| chr2 | 21022764 | 21023014 | *APOB* | + | 21022764 | 21022800 |
| chr2 | 21022847 | 21023097 | *APOB* | + | 21022847 | 21022880 |
| chr2 | 21022872 | 21023122 | *APOB* | - | 21023094 | 21023122 |
| chr2 | 21023461 | 21023711 | *APOB*;_ENSG00000280390_ | + | 21023461 | 21023497 |
| chr2 | 21023563 | 21023813 | *APOB*;_ENSG00000280390_ | + | 21023563 | 21023599 |
| chr2 | 21024668 | 21024918 | *APOB* | + | 21024668 | 21024711 |
| chr2 | 21024759 | 21025009 | *APOB* | + | 21024759 | 21024795 |
| chr2 | 21024802 | 21025052 | *APOB* | - | 21025028 | 21025052 |
| chr2 | 21025026 | 21025176 | *APOB* | - | 21025147 | 21025176 |
| chr2 | 21026728 | 21026978 | *APOB* | + | 21026728 | 21026761 |
| chr2 | 21026777 | 21027027 | *APOB* | - | 21026994 | 21027027 |
| chr2 | 21027761 | 21028011 | *APOB* | + | 21027761 | 21027796 |
| chr2 | 21027778 | 21028028 | *APOB* | - | 21027993 | 21028028 |
| chr2 | 21027887 | 21028137 | *APOB* | - | 21028101 | 21028137 |
| chr2 | 21028251 | 21028501 | *APOB* | + | 21028251 | 21028281 |
| chr2 | 21028329 | 21028579 | *APOB* | + | 21028329 | 21028365 |
| chr2 | 21028354 | 21028604 | *APOB* | - | 21028576 | 21028604 |
| chr2 | 21029565 | 21029815 | *APOB* | + | 21029565 | 21029600 |
| chr2 | 21029817 | 21030067 | *APOB* | + | 21029817 | 21029843 |
| chr2 | 21029615 | 21029865 | *APOB* | - | 21029832 | 21029865 |
| chr2 | 21029890 | 21030140 | *APOB* | + | 21029890 | 21029926 |
| chr2 | 21032253 | 21032503 | *APOB* | + | 21032253 | 21032288 |
| chr2 | 21032273 | 21032523 | *APOB* | - | 21032499 | 21032523 |
| chr2 | 21032389 | 21032639 | *APOB* | - | 21032603 | 21032639 |
| chr2 | 21033232 | 21033482 | *APOB* | + | 21033232 | 21033268 |
| chr2 | 21033320 | 21033570 | *APOB* | + | 21033320 | 21033350 |
| chr2 | 21033334 | 21033584 | *APOB* | - | 21033546 | 21033584 |
| chr2 | 21034720 | 21034970 | *APOB* | + | 21034720 | 21034748 |
| chr2 | 21034723 | 21034973 | *APOB* | - | 21034936 | 21034973 |
| chr2 | 21035457 | 21035707 | *APOB* | - | 21035683 | 21035707 |
| chr2 | 21035530 | 21035780 | *APOB* | - | 21035743 | 21035780 |
| chr2 | 21037016 | 21037266 | *APOB* | + | 21037016 | 21037048 |
| chr2 | 21037058 | 21037308 | *APOB* | - | 21037280 | 21037308 |
| chr2 | 21037889 | 21038139 | *APOB* | + | 21037889 | 21037925 |
| chr2 | 21037922 | 21038172 | *APOB* | - | 21038136 | 21038172 |
| chr2 | 21040871 | 21041121 | *APOB* | + | 21040871 | 21040900 |
| chr2 | 21040888 | 21041138 | *APOB* | - | 21041106 | 21041138 |
| chr2 | 21042273 | 21042523 | *APOB* | + | 21042273 | 21042293 |
| chr2 | 21042282 | 21042532 | *APOB* | - | 21042502 | 21042532 |
| chr2 | 21043444 | 21043694 | *APOB* | + | 21043444 | 21043467 |
| chr2 | 21043698 | 21043948 | *APOB* | - | 21043930 | 21043948 |
| chr2 | 21043802 | 21044052 | *APOB* | - | 21044029 | 21044052 |
